# Comparison of patellar tendon and hamstring grafts in ACL reconstruction: patellar tendon shows lower re-rupture rates in high-risk groups and comparable patient-reported outcomes in lower-risk patients

**DOI:** 10.1007/s00402-026-06196-5

**Published:** 2026-02-02

**Authors:** Kazumi Goto, Eisaburo Honda, Hiroshi Iwaso, Shin Sameshima, Miyu Inagawa, Yutaro Ishida, Koji Matsuo, Ryota Kuzuhara, Takaki Sanada

**Affiliations:** Department of Sports Orthopedic Surgery, Kanto Rosai Hospital, Kawasaki, Japan

**Keywords:** Anterior cruciate ligament reconstruction, Risk factor, Posterior tibial slope, Bone-patellar tendon-bone, Hamstring autograft

## Abstract

**Purpose:**

To compare re-rupture rates and clinical outcomes between bone–patellar tendon–bone (BTB) and hamstring tendon (HT) grafts in anterior cruciate ligament (ACL) reconstruction according to patient risk levels.

**Methods:**

This retrospective cohort study included patients who underwent primary ACL reconstruction at a single institution between 2018 and 2022, with outcomes assessed at a fixed 2-year postoperative follow-up. The primary endpoint was graft re-rupture, defined as a traumatic graft failure confirmed clinically and by magnetic resonance imaging, and the secondary endpoint was the Knee injury and Osteoarthritis Outcome Score (KOOS). High-risk status was defined as the presence of all three risk factors: age ≤ 20 years, posterior tibial slope. (PTS) ≥ 12°, and participation in pivoting sports. For patients with two or fewer risk factors, propensity score matching was performed using age, sex, body weight, generalized joint laxity, knee hyperextension, participation in pivoting sports, and PTS as covariates to compare outcomes between BTB and HT grafts.

**Results:**

In the high-risk group, the BTB graft showed a significantly lower re-rupture rate compared to the HT graft (12.9% vs. 35.7%, *p* = 0.03), with no significant difference in KOOS overall score (96.3 ± 3.7 vs. 96.6 ± 6.3, *p* = 0.85). In the lower-risk group after matching, the BTB graft showed a similar re-rupture rate compared to the HT graft (6.9% vs. 5.2%, *p* = 0.99), with no significant difference in KOOS overall score (92.6 ± 6.9 vs. 94.8 ± 5.7, *p* = 0.10).

**Conclusion:**

BTB grafts reduced re-rupture rates compared to HT grafts in high-risk ACL reconstruction patients, while clinical outcomes were similar. In lower-risk patients, no significant differences were observed between graft types in either re-rupture rates or KOOS overall score.

**Level of evidence:**

Level III.

**Supplementary Information:**

The online version contains supplementary material available at 10.1007/s00402-026-06196-5.

## Introduction

Recent high-quality meta-analyses and national registry-based big data studies have established a consensus that bone patellar-tendon bone (BTB) autografts result in lower re-rupture rates compared to hamstring tendon (HT) autografts in primary anterior cruciate ligament (ACL) reconstruction [1, 2]. Although some studies report no statistically significant difference in re-rupture rates between BTB and HT grafts, most show consistently lower re-rupture rates with BTB [3–5]. This may be attributed to the stronger and faster graft incorporation of BTB compared to HT, as well as the intrinsic biomechanical properties of the graft itself [6]. Recently, BTB grafts have been increasingly favored over HT grafts in high-risk patients, such as those with generalized joint laxity (GJL) or younger age [7]. This preference may be supported by evidence that patients with GJL had a fourfold higher revision risk when HT grafts were used, compared to BTB grafts [8]. However, several meta-analyses and randomized studies have reported comparable patient-reported outcomes and return-to-sport rates between BTB and HT grafts [3, 4]. These seemingly conflicting findings suggest that graft selection may not exert a uniform effect across all patients, and that a risk-profile–based approach could help reconcile these differences by identifying subgroups that may benefit from specific graft types. Despite growing attention to individualized graft selection based on risk assessment, no studies have directly compared re-rupture rates and clinical outcomes between these graft types according to patient risk profiles.

The primary objective of this study was to compare re-rupture rates and clinical outcomes between BTB and HT autografts across different risk levels, in order to clarify differences in outcomes based on patient risk profiles. As a secondary objective, re-rupture rates and outcomes were compared between the two groups after adjusting for confounding factors, allowing for a comparison between patients with similar baseline characteristics. It was hypothesized that BTB grafts would result in lower re-rupture rates in high-risk patients, whereas no significant differences in re-rupture rates or clinical outcomes would be observed between the two graft types after adjustment for risk factors.

## Materials and methods

### Patient selection

This retrospective cohort study was approved by the institutional review board. Patients who underwent primary ACL reconstruction using either HT or BTB autografts between 2018 and 2022 were reviewed. Inclusion criteria were a minimum follow-up of two years. The exclusion criteria were as follows: previous ipsilateral knee injuries, osteoarthritis greater than Kellgren-Lawrence grade 3, contralateral ACL injuries during follow-up, and grade 3 concomitant collateral ligament injuries.

### Surgical indications and technique

ACL reconstruction was performed in patients diagnosed with primary ACL injury who either wished to return to sports or experienced instability that interfered with activities of daily living. The choice of graft type (HT or BTB) was determined by multiple operating surgeons based on individual preferences and patient-specific factors. For ACL reconstruction using HT, a double-bundle technique was employed. Femoral tunnels were primarily created using the modified transtibial technique [9], while the transportal or outside-in technique was also applied depending on the case. For ACL reconstruction using BTB grafts, femoral tunnels were created using the transportal technique with a rectangular method [10]. Graft fixation involved a suspensory device on the femoral side, while either a button or a post screw was used on the tibial side, depending on the surgeon’s preference. Using a tensioning device (Arthrex, Naples, FL, USA), the graft was fixed in a fully extended knee position with a tension ranging from 60 to 80 N. The tension was set to the level deemed optimal for the patient and confirmed intraoperatively with the pivot-shift test [11]. A standardized post-operative rehabilitation protocol was applied to all patients [11]. In total, 19 surgeons were involved in the procedures. Because the surgical technique and graft choice could vary among surgeons, we additionally performed a mixed-effects logistic regression analysis with surgeon as a random effect to account for potential clustering (Supplementary Table 1).

### Outcome measurements and MCID

The following demographic and clinical parameters were extracted from medical records: age, sex, height, weight, laterality, time from injury to surgery, pre-operative sports type, pre-operative Tegner Activity Scale, presence of meniscal injury, knee hyperextension of 10°, GJL, and posterior tibial slope angle (PTS). GJL was evaluated using an 8-level scoring system (0–7) based on The University of Tokyo joint laxity test [12]. PTS was measured on medial tibial slopes using radiographs, following the method described in previous research [11]. Pre-operative sports type was categorized as pivoting or non-pivoting according to prior studies [13].

The primary outcome was defined as the presence or absence of re-rupture at two years postoperatively. Secondary outcomes included the evaluation of anteroposterior laxity using arthrometry, the Knee Injury and Osteoarthritis Outcome Score (KOOS) [14, 15], and the Anterior Cruciate Ligament-Return to Sport Injury (ACL-RSI) scale [16]. Anteroposterior laxity was measured using the digital arthrometer device (Kneelax3; Monitored Rehab Systems, Haarlem, The Netherlands) by determining the difference between the surgical and healthy sides at 132 N with the knee flexed at 30° [11].

To assess clinical relevance, the minimal clinically important difference (MCID) for total KOOS score was calculated using a distribution-based method: one-half of the standard deviation (1/2 SD) of the change scores (Δ). The proportion of patients exceeding these thresholds was calculated for each group.

### Definition of graft re-rupture

Graft re-rupture was defined as a traumatic episode with an obvious re-injury event that was clinically confirmed by manual examination and corroborated by MRI findings. To avoid overestimating failure, we did not use an arthrometer-based laxity threshold. Arthroscopic findings incidentally observed during second-look procedures were not considered, to ensure a consistent definition of re-rupture. Only patients who were followed up at our institution were included; cases in which re-rupture was confirmed at other institutions were excluded. Therefore, re-rupture was assessed at the time of the scheduled 2-year follow-up or when a distinct re-injury occurred within the 2-year period. Because of the retrospective design, blinding of the evaluators was not feasible.

Risk Factors.

Based on our previous research, age ≤ 20 years, PTS ≥ 12 degrees, and preoperative participation in pivoting sports were hypothesized as risk factors [13]. To validate the accuracy of this risk definition, logistic regression analysis was performed by including four additional factors: Graft type (BTB or HT), GJL, sex, and hyperextension of the knee, along with the original three risk factors. These additional variables were selected based on previous literature [17] reporting them as potential but controversial risk factors for graft re-rupture, with the aim of examining their significance within our cohort. As a result, the only significant independent risk factors were age ≤ 20 years, PTS ≥ 12 degrees, and preoperative participation in pivoting sports, consistent with previous findings [13]. Therefore, these three factors were adopted as the risk criteria (Table 1). Sensitivity analyses were performed to evaluate the robustness of the PTS threshold. Logistic regression models were repeated using alternative cut-off values of 10° and 11°. The association between steeper PTS and graft re-rupture remained consistent across all models.

**Table 1:** Results of the logistic regression analysis

Variable	Odds Ratio	95% CI Lower	95% CI Upper	*P*-value
(Intercept)	< 0.01	0	Inf	0.99
Sex (male)	1.01	0.44	2.30	0.98
HT graft	1.29	0.51	5.79	0.59
GJL	1.05	0.82	1.35	0.68
Hyperextension	< 0.01	0	Inf	0.99
Pivoting sports	3.47	1.16	10.4	0.03
PTS ≥ 12°	2.62	1.23	5.57	0.01
Age ≤ 20 years	2.59	1.16	5.79	0.02

Generalized joint laxity (GJL) was scored using the University of Tokyo test (scores: 0–7) [12]

*CI* confidence intervals; *HT* hamstring tendon; *PTS* posterior tibial slope

**Fig. 1:**
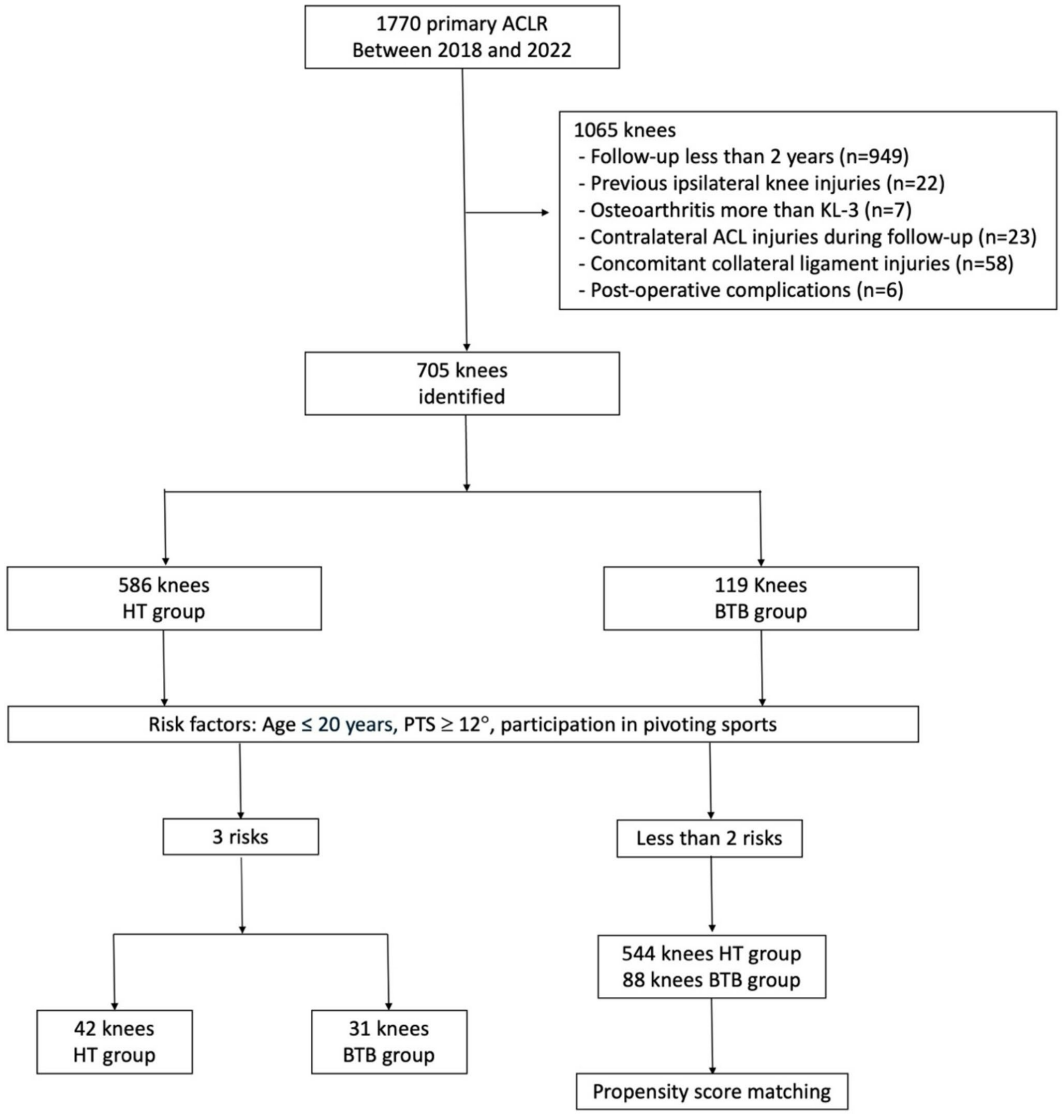
Flowchart illustrating the criteria for inclusion in and exclusion from the study. *ACL* anterior cruciate ligament; *BTB* bone patellar-tendon bone; *HT* hamstring tendon; *KL* Kellgren-Lawrence grading; *PTS* posterior tibial slope

**Fig. 2:**
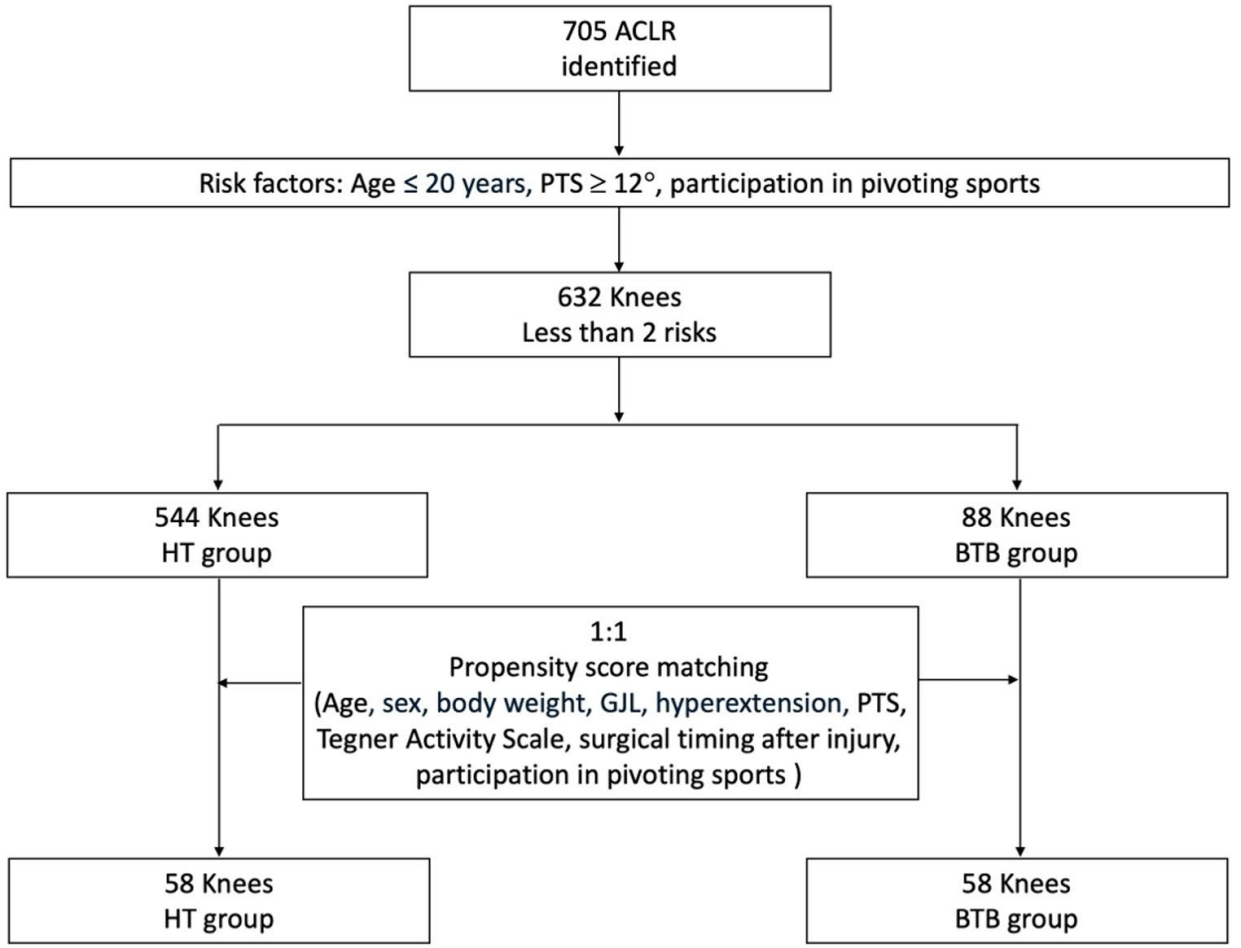
Flowchart of propensity score matching. *ACLR* anterior cruciate ligament reconstruction; *BTB* bone patellar-tendon bone; *HT* hamstring tendon; *KL* Kellgren-Lawrence grading; *PTS* posterior tibial slope

Patients with all three risk factors were defined as the high-risk group, and outcomes were compared between BTB and HT grafts within this group (Fig. 1). Additionally, for cases with two or fewer risk factors, propensity scores were calculated using five variables—age ≤ 20 years, PTS ≥ 12 degrees, preoperative participation in pivoting sports, surgical timing after injury, and preoperative Tegner Activity Scale—which had shown significant between-group differences in baseline characteristics, and 1:1 matching was performed. Outcomes were then compared between the BTB and HT graft groups (Fig. 2). In addition to the primary analysis, unadjusted and adjusted logistic regression analyses were performed for both the overall cohort and the high-risk subgroup, defined as patients aged ≤ 20 years who participated in pivoting sports and had a PTS ≥ 12°. Odds ratios (ORs) with 95% confidence intervals (CIs) were calculated, using HT graft as the reference category.

### Statistical analyses

All statistical analyses were performed using R software (version 4.2.1; R Development Core Team, Vienna, Austria). Mean values and standard deviations were used for continuous variables that showed a normal distribution, whereas medians and interquartile ranges were used for continuous variables that did not show a normal distribution. We used Student’s t-tests, Mann–Whitney U tests, and chi-square tests to compare means, medians, and nominal variables, respectively, when comparing clinical outcomes, demographic variables, pre-operative variables, and intraoperative variables between the groups.

To evaluate clinically meaningful improvement, the MCID for total KOOS score was calculated using a distribution-based method. Specifically, the MCID threshold was defined as one-half of the standard deviation (1/2 SD) of the change score (Δ = postoperative – preoperative). The proportion of patients who met or exceeded this threshold was determined for each group and used for between-group comparisons.

A post hoc power analysis was performed to assess whether the sample size was sufficient to detect differences in the primary outcome (re-rupture rate) between the BTB and HT groups. Assuming an alpha level of 0.05 and the observed re-rupture rates in the high-risk group (12.9% for BTB and 35.7% for HT), the power to detect a statistically significant difference was 99.7%, indicating that the sample size in this subgroup was adequate.

Propensity score matching was performed using a nearest-neighbor algorithm without replacement and a 1:1 matching ratio. Covariates included age, sex, body weight, GJL, knee hyperextension, PTS, and participation in pivoting sports. Balance between the BTB and HT groups before and after matching was assessed using standardized mean differences (SMDs), with an absolute SMD < 0.1 considered indicative of excellent covariate balance. The distribution of propensity scores (“distance”) was visualized using a Love plot (Supplementary Fig. 1), and detailed SMD values are summarized. Forest plots were generated to visualize the adjusted odds ratios and 95% confidence intervals for graft re-rupture in the overall cohort, the predefined high-risk subgroup, and the propensity score–matched cohort.

## Results

A total of 1,770 cases were screened. After applying exclusion criteria, 705 patients met the eligibility requirements and were included in the analysis (Fig. 1). The demographic data are summarized in Table 2. Overall, re-ruptures were observed in 37 out of 586 cases (6.3%) in the HT group and 12 out of 119 cases (10.1%) in the BTB group. In the high-risk group, the re-rupture rate was 15 out of 42 cases (35.7%) in the HT group and 4 out of 31 cases (12.9%) in the BTB group, with a statistically significant difference (*p* = 0.03). Unadjusted and adjusted analyses for the overall and high-risk subgroups are summarized in Supplementary Table 2. Sensitivity analyses using different PTS thresholds (10°, 11°, and 12°) yielded consistent results (Supplementary Table 3).

The anteroposterior laxity difference between the surgical and healthy sides was 1.6 ± 2.2 mm in the hamstring tendon group and 0.7 ± 1.4 mm in the BTB group (*p* = 0.08). No statistically significant differences were observed in KOOS or ACL-RSI scores between the two groups at two years postoperatively (Table 3). The proportion of patients exceeding the MCID threshold for total KOOS score was also similar between the groups in the high-risk cohort (*p* = 0.60).

**Table 2:** Patient demographics

	HT (*n* = 586)	BTB (*n* = 119)	*p*-value
Age (years)	29.3 ± 13.0	20.6 ± 7.6	< 0.01
Sex (female/male)	324/262	61/58	0.08
Left/right	295/291	61/58	0.94
Height (cm)	165.4 ± 8.5	166.3 ± 9.4	0.29
Body weight (kg)	62.6 ± 11.8	67.2 ± 18.4	< 0.01
Surgical timing after injury (days)	75 (48–152)	52 (38–87)	< 0.01
GJL	1 (0–7)	2 (0–7)	< 0.01
Hyperextension knee	62 (11.8%)	20 (18.0%)	0.25
Tegner Activity Scale	6.6 ± 1.2	7.7 ± 1.5	< 0.01
Pivoting sports	346 (59.2%)	79 (66.4%)	< 0.01
MM injury	190 (32.4%)	25 (21.0%)	0.02
LM injury	195 (33.3%)	36 (30.3%)	0.59
Posterior tibial slope angle (°)	10.1 ± 3.0	11.5 ± 3.0	< 0.01

Generalized joint laxity (GJL) was scored using the University of Tokyo test (scores: 0–7) [12]

*HT* hamstring tendon: *BTB* bone-patellar tendon-bone; *MM* medial meniscus; *LM* lateral meniscus

**Table 3:** Comparison results in high-risk cases (patients with all three factors: age ≤ 20 years, PTS ≥ 12°, and participation in Pivoting sports)

		HT (*n* = 42)	BTB (*n* = 31)	*p*-value
Re-rupture, n (%)		15 (35.7%)	4 (12.9%)	0.03
Age (years)		15.7 ± 1.5	16.7 ± 1.6	< 0.01
Sex (female/male)		24/18	12/19	0.16
Left/right		20/22	16/15	0.82
Height (cm)		165.0 ± 9.2	167.9 ± 7.5	0.15
Body weight (kg)		57.2 ± 9.7	62.7 ± 11.0	0.03
Tegner Activity Scale		7.1 ± 0.6	7.8 ± 1.1	< 0.01
2-year KOOS	Symptom	95.8 ± 6.0	93.0 ± 7.5	0.20
	Pain	97.1 ± 5.4	97.5 ± 3.8	0.78
	ADL	99.2 ± 3.1	99.6 ± 1.4	0.64
	Sports	92.8 ± 16.7	94.0 ± 8.7	0.76
	QOL	91.2 ± 16.9	88.8 ± 13.4	0.61
	Overall	96.6 ± 6.3	96.3 ± 3.7	0.85
∆KOOS (overall)		14.8 ± 11.1	13.3 ± 8.0	0.69
MCID (KOOS overall)		71.4%	77.4%	0.60
2-year ACL-RSI score		77.2 ± 21.1	68.7 ± 15.3	0.20

Data are expressed as means ± standard deviations, and n (%)

*HT* hamstring tendon: *BTB* bone-patellar tendon-bone; 2-year KOOS, the 2-year postoperative Knee Injury and Osteoarthritis Outcome Score; *MCID* minimal clinically important difference; *ACL-RSI* Anterior Cruciate Ligament-Return to Sport Injury

After propensity score matching, all covariates were well balanced between the BTB and HT groups, with all absolute standardized mean differences below 0.2 (Supplementary Table 4, Supplementary Fig. 1). Re-ruptures occurred in 5 out of 73 cases (6.8%) in the HT group and 6 out of 73 cases (8.2%) in the BTB group, with no statistically significant difference (Table 4). The anteroposterior laxity difference between the surgical and healthy sides was 1.0 ± 1.6 mm in the HT group and 0.9 ± 1.5 mm in the BTB group (*p* = 0.81). No significant differences were found in KOOS or ACL-RSI scores between the two groups at two years postoperatively (Table 4). Similarly, the proportion of patients exceeding the MCID threshold for total KOOS score did not differ significantly between the groups after matching (*p* = 0.99).

**Table 4:** Demographic data before and after propensity score matching

		Pre-matching			Post-matching		
		HT (*n* = 544)	BTB (*n* = 88)	*p*-value	HT (*n* = 58)	BTB (*n* = 58)	*p*-value
Age (years)		30.3 ± 12.8	22.0 ± 8.3	< 0.01	22.1 ± 9.5	22.5 ± 9.4	0.62
Sex (female/male)		300/244	49/39	0.08	37/21	38/20	0.99
Left/right		275/269	45/43	0.96	27/31	30/28	0.71
Height (cm)		165.5 ± 8.4	165.8 ± 9.9	0.75	163.6 ± 8.5	163.3 ± 8.7	0.86
Body weight (kg)		62.9 ± 11.8	68.8 ± 20.1	< 0.01	62.1 ± 13.9	62.3 ± 13.8	0.88
Surgical timing after injury (days)		76 (48–152)	52 (38–87)	< 0.01	66 (41–102)	56 (38–125)	0.53
Generalized joint laxity		1 (0–2)	2 (0–3)	0.06	2 (1–3)	2 (0–3)	0.39
Hyperextension knee		59 (12.1%)	15 (18.3%)	0.28	14 (24.1%)	12 (20.7%)	0.82
Tegner Activity Scale		6.5 ± 1.2	7.6 ± 1.7	< 0.01	7.0 ± 1.1	7.1 ± 1.6	0.23
Pivoting sports		304 (56.1%)	48 (54.5%)	0.82	34 (58.6%)	33 (56.9%)	0.99
MM injury		183 (33.6%)	18 (20.5%)	0.10	18 (31.0%)	13 (22.4%)	0.40
LM injury		184 (33.8%)	28 (31.8%)	0.81	21 (36.2%)	17 (29.3%)	0.55
Posterior tibial slope (°)		9.9 ± 3.0	10.7 ± 3.1	< 0.01	10.3 ± 2.9	10.2 ± 3.0	0.78
Re-rupture, n (%)		22 (4.0%)	8 (9.1%)	0.054	3 (5.2%)	4 (6.9%)	0.99
2-year KOOS	Symptom	90.4 ± 9.6	90.4 ± 9.9	0.98	93.1 ± 7.7	90.3 ± 10.3	0.28
	Pain	94.7 ± 7.4	94.2 ± 6.4	0.64	96.6 ± 6.1	94.1 ± 6.4	0.02
	ADL	98.3 ± 5.9	99.1 ± 1.8	0.29	99.6 ± 1.1	99.2 ± 1.4	0.07
	Sports	91.6 ± 12.9	89.5 ± 12.2	0.23	96.3 ± 5.8	90.7 ± 10.0	0.01
	QOL	84.9 ± 16.6	85.9 ± 16.6	0.65	88.3 ± 17.1	88.9 ± 12.6	0.59
	Overall	93.9 ± 7.3	94.2 ± 5.5	0.79	94.8 ± 5.7	92.6 ± 6.9	0.10
∆KOOS (overall)		18.9 ± 13.2	13.6 ± 9.6	0.01	18.0 ± 12.5	13.1 ± 8.0	0.15
MCID (KOOS overall)		85.9%	82.2%	0.50	90.6%	86.1%	0.70
2-year ACL-RSI score		63.7 ± 22.6	67.2 ± 19.0	0.35	72.4 ± 23.8	66.6 ± 17.2	0.13

Data are expressed as means ± standard deviations, medians (interquartile ranges), and n (%)

Generalized joint laxity was scored using the University of Tokyo test (scores: 0–7) [14]

*HT* hamstring tendon: *BTB* bone-patellar tendon-bone; *MM* medial meniscus; *LM* lateral meniscus; 2-year KOOS, the 2-year postoperative Knee Injury and Osteoarthritis Outcome Score; *MCID* minimal clinically important difference; *ACL-RSI* Anterior Cruciate Ligament-Return to Sport Injury

**Fig. 3:**
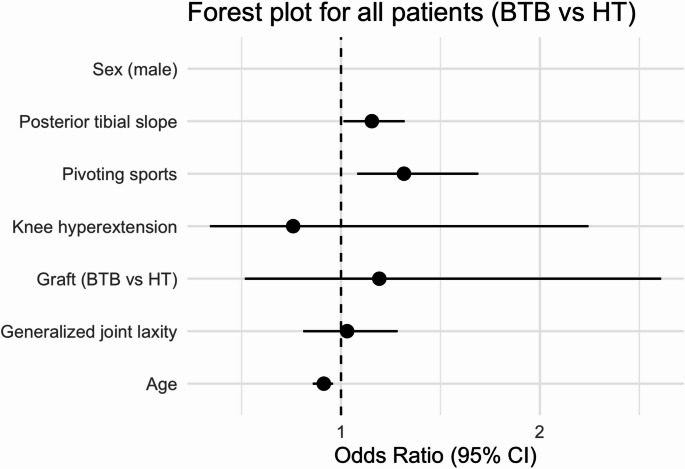
Forest plot showing adjusted odds ratios (95% CIs) for graft re-rupture in the overall cohort. BTB, bone patellar-tendon bone; CI, confidence interval; HT, hamstring tendon

**Fig. 4:**
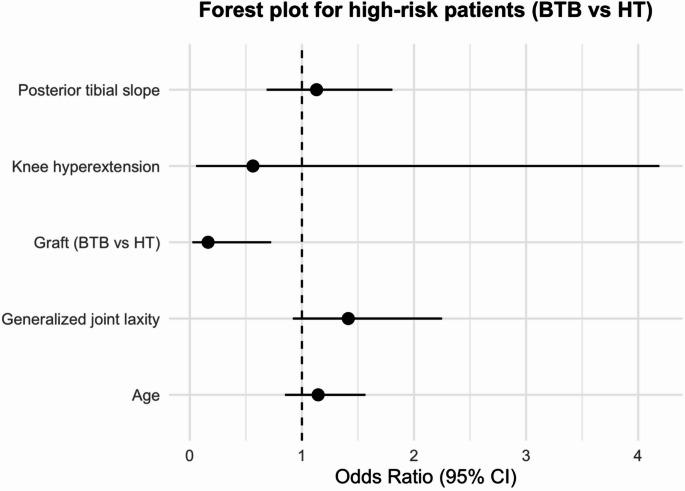
Forest plot showing adjusted odds ratios (95% CIs) for graft re-rupture in the high-risk subgroup (age ≤ 20 years, PTS ≥ 12°, and pivoting sports). *BTB* bone patellar-tendon bone; *CI* confidence interval; *HT* hamstring tendon

**Fig. 5:**
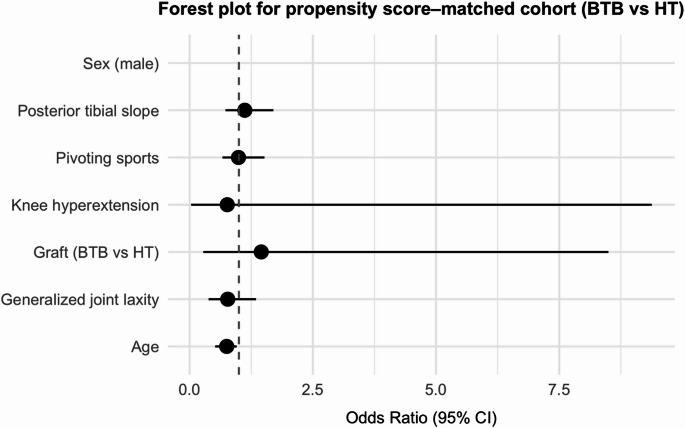
Forest plot showing adjusted odds ratios (95% CIs) for graft re-rupture in the propensity score–matched cohort. *BTB* bone patellar-tendon bone; *CI* confidence interval; *HT* hamstring tendon

Forest plots summarizing adjusted odds ratios (ORs) for graft re-rupture are shown in Figs. 3, 4 and 5. In the overall cohort (Fig. 3), younger age, steeper PTS, and participation in pivoting sports were independently associated with higher re-rupture risk, whereas graft type (BTB vs. HT) was not significant. In the predefined high-risk subgroup (Fig. 4), BTB graft use was associated with lower odds of re-rupture (the only significant predictor), while other covariates were not significant. In the propensity score–matched cohort (Fig. 5), no variable reached statistical significance.

## Discussion

The most important finding in this study was that in patients with all three risk factors—age ≤ 20 years, PTS ≥ 12°, and participation in pivoting sports—the re-rupture rate at two years postoperatively was significantly lower in the BTB graft group compared to the HT graft group. However, after adjusting for confounding factors, no significant difference in re-rupture rates was observed between the two graft types. Additionally, in both the high-risk group and the propensity score-matched cohort, no significant differences were found in the overall KOOS or ACL-RSI scores between the two groups at two years postoperatively. Nevertheless, when examining individual KOOS subscales in the low-risk matched cohort, the BTB group showed significantly lower scores in the pain and sports domains. Based on these results, it may be advisable for surgeons concerned about re-rupture risk to consider BTB grafts over HT grafts in high-risk patients, whereas HT grafts might be preferable for lower-risk patients from a symptom and comfort perspective.

Numerous studies have compared BTB and HT grafts, with a consensus that BTB grafts result in lower re-rupture rates compared to HT grafts [4, 5, 18]. Although some studies have reported no significant difference in re-rupture rates between HT and BTB grafts [1, 2], a recent analysis using the Reverse Fragility Index —a novel metric for assessing the robustness of studies reporting non-significant results—revealed that many randomized controlled trials reporting no significant difference were vulnerable to statistical instability. Specifically, the reversal of outcomes in a small number of patients could lead to a significant difference, and in many cases, this number was smaller than the dropout rate, indicating that these findings may be fragile [19]. Although comparative studies of BTB and HT grafts focusing on high-risk patients, such as young females or highly active individuals, have been conducted [10, 20, 21], no studies have simultaneously adjusted for multiple confounding factors, such as PTS, age, and sex, to compare BTB and HT grafts. Consistent with previous research, our results showed that in high-risk patients, the re-rupture rate was lower with BTB grafts compared to HT grafts.

Large national registry studies have consistently shown lower revision rates with BTB grafts compared with hamstring grafts in the general ACL reconstruction population [4, 5, 18]. Our findings are in line with this overall trend but further refine it by identifying a specific high-risk triad—age ≤ 20 years, PTS ≥ 12°, and participation in pivoting sports—in which the BTB advantage is particularly pronounced. This pattern may be explained by the greater initial fixation strength, bone-to-bone healing, and reduced graft elongation associated with BTB grafts, which are especially beneficial under high mechanical stress [6–8]. However, registry-level results must be interpreted with caution because of inherent selection bias. Surgeons tend to choose BTB grafts for younger or higher-risk athletes [7, 8], resulting in an overrepresentation of high-risk cases in the BTB group. Consequently, unadjusted comparisons may show similar re-rupture rates with BTB grafts [1, 2], although this likely reflects the higher baseline risk of the BTB population rather than inferior graft performance.

On the other hand, BTB grafts are associated with issues such as anterior knee pain (AKP) and reduced quadriceps strength during extension [22, 23]. A recent study using New Zealand’s national registry also reported that while there was no significant difference in the KOOS pain subscore between HT and BTB grafts, the rate of severe kneeling difficulty was significantly higher in the BTB group (21.3%) compared to the HT group (9.4%) [24]. Given these BTB-specific complications, it is inappropriate to choose BTB grafts solely due to their lower re-rupture rate and comparable KOOS scores to HT grafts, without considering other important factors. In the present study, when additional covariates such as GJL, sex, body weight, and knee hyperextension were included in the propensity score matching, the KOOS-pain and KOOS-sports subscales became significantly lower in the BTB group than in the HT group. This finding suggests that donor-site morbidity, particularly anterior knee pain, may have influenced these results. Accordingly, while BTB grafts may reduce the risk of re-rupture in high-risk populations, hamstring grafts could be more favorable for lower-risk patients in terms of patient-reported outcomes and overall comfort. Particularly, based on our findings, re-rupture rates and KOOS scores were comparable in patients with two or fewer risk factors. Therefore, for such patients, it is important to consider the patient’s background—including the type of sport, psychological state, and preoperative muscle strength—when selecting the most appropriate graft [6, 25]. In this context, graft selection should be approached through a shared decision-making process, in which surgeons discuss not only the potential benefits of each graft type but also their respective trade-offs—such as the risk of donor-site morbidity or anterior knee symptoms—with the patient. Ultimately, the optimal graft choice should reflect each patient’s activity goals, symptom tolerance, and personal priorities.

This study had several limitations. First, it was retrospective in nature. Second, the choice of graft and surgical technique varied among 19 surgeons, introducing potential confounding by technique despite the use of a mixed-effects model to account for clustering. Third, the number of high-risk patients was relatively small, which may limit statistical power. Fourth, all outcomes were evaluated at a fixed 2-year postoperative follow-up; thus, long-term differences could not be assessed. Finally, BTB-specific symptoms such as AKP, kneeling difficulty, and quadriceps weakness were not directly evaluated. Despite these limitations, our findings should be interpreted as hypothesis-generating. The results suggest a possible trend that BTB grafts may reduce re-rupture risk in high-risk patients, whereas clinical outcomes were otherwise comparable between graft types. Further prospective studies with larger and longer follow-up cohorts are needed to confirm these observations.

The clinical relevance of this study lies in proposing a potential strategy for graft selection based on individual risk profiles, thereby contributing to more personalized decision-making in ACL reconstruction.

## Conclusion

In high-risk cases with an age of ≤ 20 years, PTS of ≥ 12°, and participation in pivoting sports, the re-rupture rate was significantly lower in the BTB group compared to the HT group, while clinical outcomes were comparable. However, after adjusting for risk factors, no significant differences were observed between the two groups in terms of re-rupture rates or clinical outcomes.

## Supplementary Information

Below is the link to the electronic supplementary material.

Supplementary Material 1

Supplementary Material 2

Supplementary Material 3

Supplementary Material 4

Supplementary Material 5

## Data Availability

The datasets used and/or analysed during the current study are available from the corresponding author on reasonable request.
