# Supplementary material for: Comparison of patellar tendon and hamstring grafts in ACL reconstruction: patellar tendon shows lower re-rupture rates in high-risk groups and comparable patient-reported outcomes in lower-risk patients
Source: Arch Orthop Trauma Surg. 2026 Feb 2;146(1):51. doi: 10.1007/s00402-026-06196-5 (PMC12864351; doi:10.1007/s00402-026-06196-5)
Supplement: Supplementary file 1 — Supplementary Material 1 [file 402_2026_6196_MOESM1_ESM.docx]

**Supplementary Table 1. Mixed-effects logistic regression analysis including surgeon as a random effect**

The random intercept variance for surgeons was 1.26 (SD = 1.12), indicating moderate inter-surgeon variability.

Fixed-effect estimates are presented as log-odds coefficients with corresponding standard errors, odds ratios, and 95% confidence intervals.

| Variable | Estimate | Std. Error | Odds Ratio | 95% CI Lower | 95% CI Upper |
| --- | --- | --- | --- | --- | --- |
| (Intercept) | -2.93419 | 0.002025 | 0.053174 | 0.052963 | 0.053385 |
| BTB graft | -0.12353 | 0.002024 | 0.883799 | 0.8803 | 0.887312 |
| Age | -0.12376 | 0.002036 | 0.883595 | 0.880076 | 0.887128 |
| Sex (male) | 0.245454 | 0.002024 | 1.278202 | 1.273141 | 1.283283 |
| Pivoting sports | 0.141437 | 0.002026 | 1.151928 | 1.147364 | 1.15651 |
| PTS | 0.209979 | 0.002029 | 1.233653 | 1.228756 | 1.238569 |

Std., standard; BTB, bone-patellar tendon-bone; PTS, posterior tibial slope
