# Supplementary material for: Comparison of patellar tendon and hamstring grafts in ACL reconstruction: patellar tendon shows lower re-rupture rates in high-risk groups and comparable patient-reported outcomes in lower-risk patients
Source: Arch Orthop Trauma Surg. 2026 Feb 2;146(1):51. doi: 10.1007/s00402-026-06196-5 (PMC12864351; doi:10.1007/s00402-026-06196-5)
Supplement: Supplementary file 2 — Supplementary Material 2 [file 402_2026_6196_MOESM2_ESM.docx]

**Supplementary Table 2. Unadjusted and adjusted logistic regression analyses for overall and high-risk subgroups**

Unadjusted and adjusted logistic regression analyses for graft re-rupture in the overall cohort and the high-risk subgroup (age ≤ 20 years, PTS ≥ 12°, and participation in pivoting sports). Odds ratios (ORs) and 95% confidence intervals (CIs) are shown. In all models, the hamstring tendon graft was used as the reference category. The adjusted models included sex, generalized joint laxity (GJL), knee hyperextension, pivoting sports participation, PTS > 12°, and age ≤ 20 years as covariates.

| Model | Variable | OR | 95% CI | P value |
| --- | --- | --- | --- | --- |
| Overall (Unadjusted) | BTB graft | 1.70 | 0.81-3.31 | 0.14 |
| Overall (adjusted) | BTB graft | 1.12 | 0.45-2.57 | 0.80 |
|  | Sex (male) | 1.51 | 0.70-3/36 | 0.30 |
|  | GJL | 2.23 | 0.81-5.61 | 0.10 |
|  | Hyperextension | 0.73 | 0.38-1.85 | 0.42 |
|  | Age ≤ 20 years | 3.20 | 1.45-7.29 | 0.004 |
|  | Pivoting sports | 1.28 | 1.06-1.60 | 0.017 |
|  | PTS ≥12° | 1.45 | 1.01-2.10 | 0.044 |
| Unadjusted (High-risk: U-20 + Pivoting + PTS>12) | BTB graft | 0.35 | 0.09-1.08 | 0.086 |
| Adjusted (High-risk: U-20 + Pivoting + PTS>12) | BTB graft | 0.15 | 0.02-0.78 | 0.047 |
|  | Sex (male) | 1.12 | 0.27-4.90 | 0.876 |
|  | GJL | 16.0 | 2.23-187.5 | 0.011 |
|  | Hyperextension | 0.34 | 0.02-3.02 | 0.372 |

BTB, bone-patellar tendon-bone; PTS, posterior tibial slope
