# Supplementary material for: Comparison of patellar tendon and hamstring grafts in ACL reconstruction: patellar tendon shows lower re-rupture rates in high-risk groups and comparable patient-reported outcomes in lower-risk patients
Source: Arch Orthop Trauma Surg. 2026 Feb 2;146(1):51. doi: 10.1007/s00402-026-06196-5 (PMC12864351; doi:10.1007/s00402-026-06196-5)
Supplement: Supplementary file 3 — Supplementary Material 3 [file 402_2026_6196_MOESM3_ESM.docx]

**Supplementary Table 3. Sensitivity analyses using alternative PTS thresholds.**

Logistic regression analyses were performed using PTS cut-offs of 10°, 11°, and 12°.

Higher PTS was consistently associated with increased risk of graft re-rupture across all thresholds.

| Model | Variable | OR | 95% CI | P value |
| --- | --- | --- | --- | --- |
| PTS ≥ 10° | PTS10 | 2.25 | 1.14-4.79 | 0.026 |
| PTS ≥ 11° | PTS11 | 2.60 | 1.36-5.31 | 0.0048 |
| PTS ≥ 12° | PTS12 | 3.38 | 1.81-6.51 | 0.00018 |

OR, odds ratio; CI,; PTS, posterior tibial slope
