# Supplementary material for: Comparison of patellar tendon and hamstring grafts in ACL reconstruction: patellar tendon shows lower re-rupture rates in high-risk groups and comparable patient-reported outcomes in lower-risk patients
Source: Arch Orthop Trauma Surg. 2026 Feb 2;146(1):51. doi: 10.1007/s00402-026-06196-5 (PMC12864351; doi:10.1007/s00402-026-06196-5)
Supplement: Supplementary file 4 — Supplementary Material 4 [file 402_2026_6196_MOESM4_ESM.docx]

**Supplementary Table 4. Sensitivity analyses using alternative PTS thresholds.**

Standardized mean differences (SMD) of covariates before and after propensity score matching between the bone-patellar tendon-bone and hamstring tendon groups. Adequate balance was defined as an absolute SMD < 0.1, with values < 0.2 considered acceptable.

| Variable | SMD before | SMD after |
| --- | --- | --- |
| Distance | 0.844 | 0.025 |
| Age | -1.046 | 0.054 |
| Sex | 0.035 | -0.081 |
| BMI | 0.27 | 0.149 |
| GJL | 0.253 | -0.016 |
| Hyperextension | 0.238 | -0.167 |
| PTS | 0.174 | -0.042 |
| Pivoting sport | 0.007 | 0.054 |

BMI, bone mass index; GJL, generalized joint laxity; PTS, posterior tibial slope
